# CRT combined with a sequential VMAT boost in the treatment of upper thoracic esophageal cancer

**DOI:** 10.1120/jacmp.v14i5.4325

**Published:** 2013-09-06

**Authors:** Xiance Jin, Jinling Yi, Yongqiang Zhou, Huawei Yan, Ce Han, Congying Xie

**Affiliations:** ^1^ Department of Radiotherapy and Chemotherapy the 1st Affiliated Hospital of Wenzhou Medical University Wenzhou China

**Keywords:** volumetric‐modulated arc therapy, conformal radiotherapy, boost phase, upper thoracic esophageal cancer

## Abstract

The purpose of this study is to investigate the potential benefits of conformal radiotherapy (CRT) combined with a sequential volumetric‐modulated arc therapy (VMAT) boost in the treatment of upper thoracic esophageal cancer. Ten patients with upper thoracic esophageal cancer previously treated with CRT plus a sequential VMAT boost plan were replanned with CRT plus an off‐cord CRT boost plan and a full course of VMAT plan. Dosimetric parameters were compared. Results indicated that CRT plus off‐cord CRT boost was inferior in planning target volume (PTV) coverage, as indicated by the volume covered by 93% (p = 0.05) and 95% (p = 0.02) of the prescription dose. The full course VMAT plan was superior in conformal index (CI) and conformation number (CN), and produced the highest protection for the spinal cord. CRT plus a VMAT boost demonstrated significant advantages in decreasing the volume of the lung irradiated by a dose of 10 Gy (V10, p = 0.007),13 Gy (V13, p = 0.003), and 20 Gy (V20, p = 0.001). The full course VMAT plan demonstrated the lowest volume of lung receiving a dose of 30 Gy. CRT plus a VMAT boost for upper thoracic esophageal cancer can improve the target coverage and reduce the volume of lung irradiated by an intermediate dose. This combination may be a promising treatment technique for patients with upper thoracic esophageal cancer.

PACS number: 87.53.Kn, 87.55.x, 87.55.D, 87.55.dk

## I. INTRODUCTION

Conformal radiotherapy (CRT) is still the treatment of choice for esophageal cancer, with parallel opposed, antero‐posterior and postero‐anterior (APPA) fields for the first phase and a second phase with off‐cord lateral or oblique parallel opposed beams.^(1)^ In order to constrain the dose to spinal cord strictly within its tolerant limit, conformal plans may unavoidably compromise target coverage due to its unfavorable geometry close to spinal cord. As such, intensity‐modulated radiation therapy (IMRT) has been reported to provide superior target coverage while reducing doses to organs at risk (OAR) of cervical esophageal cancer.^(2)^ A full course of IMRT usually has not been recommended for thoracic cancer because of the widely distributed low dose levels surrounding the planning target volume (PTV).^(^ ^3^ ^,^ ^4^ ^)^ A hybrid technique that combines conformal fields with IMRT fields on a daily fraction basis for lung and esophageal cancer patients has been suggested to reduce the volume of the lung irradiated by relatively low dose levels.^(5)^

With the continued development in radiotherapy delivery techniques, volumetric‐modulated arc therapy (VMAT) has begun to replace conventional IMRT in the treatment of several cancers. Several studies have demonstrated that VMAT is able to produce dosimetric plans equivalent to those of IMRT.^6^, ^7^, ^8^ At the same time, VMAT is also able to reduce the number of monitor units and the required delivery time in comparison to IMRT.^6^, ^7^, ^8^ Comparative studies on VMAT and IMRT in the treatment of esophageal cancer also indicate that VMAT is able to achieve comparable target coverage and OAR sparing with reduced monitor units and delivery time.^(^ ^9^ ^,^ ^10^ ^)^ However, a full course VMAT plan for the treatment of esophageal cancer patients would exhibit the same problem as a full course IMRT plan. The purpose of this study is to investigate the potential benefit of applying the VMAT technique in the second boost phase instead of using off‐cord conformal beams in the treatment of upper thoracic esophageal cancer patients following the first APPA phase.

## II. MATERIALS AND METHODS

### A. Patients

Ten patients with upper thoracic esophageal cancer, who were previously treated in our department with APPA CRT beams for the first phase and followed with a single‐arc VMAT boost, were enrolled in this study. Each patient was replanned retrospectively via the Pinnacle treatment planning system (Philips, clinical version 9.2; Fitchburg, WI). Patient staging information according to the AJCC staging system (AJCC, 2002) and other relevant characteristics are summarized in Table 1.

Gross tumor volume (GTV), clinical target volume (CTV), PTV, and nodes were contoured by a physician according to the RTOG 0436 protocol.^(11)^ The GTV included the gross tumor and involved nodes as defined by diagnostic CT, oesophagogastroscopy, endoscopic ultrasound, and PET scan. The CTV was delineated with 3–5 cm superior‐inferior margins and 1 cm lateral and anterior‐posterior margins with respect to the GTV. The PTV was delineated with a 0. 5 cm margin from the CTV. For the sake of data consistence, the PTVs of this enrolled cohort patients were the same for both the initial and boost plans. The spinal cord, lung, and heart were contoured as OARs on each image.

**Table 1:** Patient staging and characteristics

*atient*	*Staging*	$\text{GTV}\;({\text{cm}}^{3})$	$\text{CTV}\;({\text{cm}}^{3})$	$\text{PTV}\;({\text{cm}}^{3})$
1	T3N1M1	25.3	195.3	303.0
2	T3N0M1	15.7	85.7	146.4
3	T3N1M1	15.4	155.5	230.3
4	T2N1M0	17.3	175.3	219.6
5	T3N1M1	19.4	97.4	167.0
6	T2N0M0	16.0	96.0	149.0
7	T4N1M1	35.8	358.8	477.9
8	T2N1M1	12.1	109.1	203.2
9	T4N1M1	34.3	342.3	513.2
10	T3N1M1	37.8	257.8	363.7

### B. Planning schemes

Three planning schemes were generated for each patient. Scheme 1 is CRT initial plus off‐cord CRT boost, scheme 2 is CRT initial plus a sequential VMAT boost, and scheme 3 is a full course of VMAT plan — that is, one VMAT plan for the whole treatment course. The prescription dose of CRT initial plan was 2 Gy × 18 fractions for a total dose of 36 Gy with APPA beams, followed by 2 Gy × 12 fractions for a total dose of 24 Gy with off‐cord conformal beams. The prescription dose of CRT plus a sequential boost VMAT was 2 Gy × 18 fractions for a total dose of 36 Gy with initial CRT APPA beams, followed by a single‐arc VMAT boost plan with a prescription of 2 Gy × 12 fractions. The prescription dose for the full course VMAT plan was 60 Gy over 30 fractions (2 Gy × 30 fractions). For VMAT plan optimization, constraint leaf motion of 0.46 cm/deg and final arc space degree of 4 were employed. A start angle of 181° and a stop angle of 180° were applied for one‐arc plans using clockwise (CW) rotation direction. The maximum constraint dose for the spinal cord was 400 cGy for the sequential VMAT boost plan, and 4500 cGy for the full course VMAT plan. The optimization objectives were set for the PTV, spinal cord, and lung for the VMAT plans. For upper thoracic esophageal cancer, only a very small part of heart was involved in the treatment field; the constraint on heart had little effect on the plan quality, so we did not include the heart in the inverse optimization.

All plans were optimized to reach clinically acceptable levels. For instance, the maximum dose for the spinal cord was less than 45 Gy, whereas the volume of the lung irradiated by a dose of 20 Gy (V20) and 30 Gy (V30) was less than 30% and 20%, respectively. For PTV coverage, 95% of PTV should be covered by 95% of the prescription dose, unless the spinal cord limit was violated.

### C. Plan evaluation and comparison

The following plan quality indices were calculated from the dose‐volume histogram (DVH) data for evaluation and comparison.

Target coverage (TC) of PTV was calculated to describe the percent volume of PTV covered by the prescription dose:

(1) $$\text{TC}=\frac{V_{T,\text{Pi}}}{V_{T}}$$

where $V_{T,\text{Pi}}$ is the target volume that was covered by the prescription isodose, which was set at 95% in this study. $V_{T}$ is the volume of the target.

A homogeneity index similar to that defined in the ICRU 62 Report was adapted for PTV to study the dose distribution homogeneity across the PTV:^(12)^

(2) $$\text{HI}=\frac{V95-V110}{V95}$$

The conformity index (CI)^(13)^ and conformation number (CN)^(14)^ were also calculated for PTV:

(3) $$\text{CI}=\frac{V_{T,\text{Pi}}}{V_{\text{Pi}}}$$

(4) $$\text{CN}=\frac{V_{T,\text{Pi}}}{V_{T}}\times \frac{V_{T,\text{Pi}}}{V_{\text{Pi}}}$$

where $V_{\text{Pi}}$ is the volume that was covered by the prescription isodose. The maximum value of CI is 1, corresponding to a perfect coverage of PTV. CN is the complementary information designed to compensate for defects in the TC and CI. The first term of Eq. (4) stands for the coverage of the target volume. The second term refers to the volume of healthy tissue receiving a dose equal to or greater than the prescribed dose. CN can take values between 0 and 1.

Radiobiological ranking indices, TCP and NTCP, were also calculated using the Niemierko and Goiten model.^(15)^ Based on the equivalent uniform dose (EUD), the tumor control probability (TCP) can be calculated by:

(5) $$\text{TCP}=\frac{1}{1+{\left[\frac{{\text{TCD}}_{50}}{\text{EUD}}\right]}^{4\gamma_{50}}}$$

where ${\text{TCD}}_{50}$ is the tumor dose required to produce 50% of TCP, and $\gamma_{50}$ is the slope of the dose response at 50% of TCP. These tumor‐specific parameters were cited from a study by Okunieff et al.^(16)^ The EUD is defined as the absorbed dose that, if homogeneously delivered to a tumor, would cause the same expected number of clonogens to survive as the actual nonhomogeneous absorbed dose distribution would. Clonogen survival is a stochastic magnitude governed by Poisson statistics, while EUD is obtained as an expected value:

(6) $$\text{EUD}={\left(\sum_{1}^{N}v_{i}D_{i}^{a}\right)}^{\frac{1}{a}}$$

where *N* is the number of voxels in the structure of interest, $D_{i}$ is the dose in the ith voxel $v_{i}$, and α is the tumor's normal tissue‐specific parameters that describe the dose‐volume effect. TCP of PTV was calculated for the purpose of plan evaluation.

In the case of normal tissue, the normal tissue complication probability (NTCP) is determined as:

(7) $$\begin{array}{l} \text{NTCP}=\frac{1}{1+{\left[\frac{{\text{TD}}_{50}}{\text{EUD}}\right]}^{4\gamma_{50}}} \end{array}$$

where ${\text{TD}}_{50}$ is the dose at which the probability of complication becomes 50% in five years, and $\gamma_{50}$ is the slope of the sigmoidal dose response curve of normal tissue at 50% complication probability. These tissue‐specific parameters were based on the Niemierko and Goiten model.^(15)^ NTCP of the spinal cord, heart, and lungs were calculated for plan evaluation.

The mean dose (Dmean) and maximum dose (Dmax) for OARs were calculated and compared. The volume of hearts irradiated by a dose of 25, 30, and 50 Gy (V25, V30, and V50, respectively) was calculated and compared. The volume of lung irradiated by a dose of 5, 10, 13, 20, and 30 Gy (V5, V10, V13, V20, and V30, respectively) was also calculated and compared.

### D. Statistical analysis

The different planning schemes were analyzed using the one‐way ANOVA method. When an overall significant difference was observed, the post hoc Turkey's test was used to determine which pairwise comparisons differed. All statistical analyses were conducted with SPSS 17.0 software. Differences were considered statistically significant when p < 0.05.

## III. RESULTS

None of the ten patients in this study suffered from pulmonary complications three months after their treatment course.

**Figure 1:**
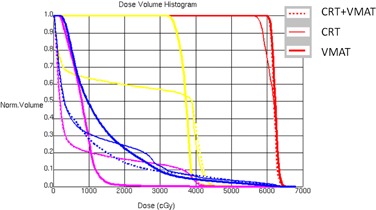
Typical dose‐volume histogram comparison among CRT plus a VMAT boost (dashed), CRT plus lateral beams (medium solid), and a full course of VMAT (thick solid). Red = PTV; yellow = spinal cord; blue = lung; and pink = heart.

**Figure 2:**
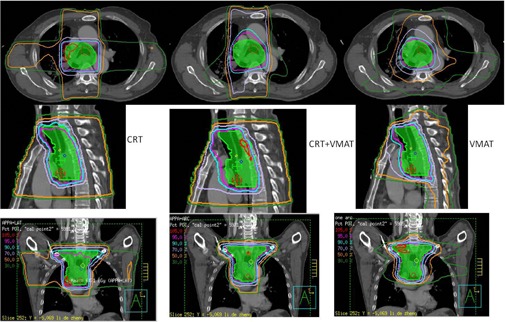
Dose distributions for CRT, CRT plus VMAT, and full course of VMAT plans in the axial, sagittal, and coronal planes.

Figure 1 shows the typical DVH data for one of the patients. In this case, CRT plus a VMAT boost and full course VMAT plan provided a better target coverage than CRT plan did. CRT plus a VMAT boost had the lowest value in terms of lung V20. Figure 2 shows the typical dose distribution of one patient. CRT plus a VMAT boost plan showed the lowest volume of healthy tissue irradiation by the 30% isodose line in the axial and coronal planes. The 95% isodose line did not cover the PTV in the CRT plans due to the tolerant dose limitation of spinal cord, which is in a close proximity to the PTV.

Average target coverage of the different treatment schemes is summarized in Table 2. The mean dose and EUD values were similar for all the treatment schemes. CRT was inferior in TC, as indicated by the volume covered by 93% (p = 0.05) and 95% (p = 0.02) of the prescription dose. The full course VMAT plan was superior in terms of CI (p = 0.01) and CN (p = 0.01). The OAR sparing comparison is summarized in Table 3. The full course VMAT plan achieved the highest protection for the spinal cord. CRT showed the lowest low dose volume (V5) for the lung, although this result was not statistically significant (p = 0.11). CRT plus a VMAT boost plan demonstrated great advantages in decreasing the volume of the lung irradiated by a dose of 10 Gy (V10, p = 0.007),13 Gy (V13, p = 0.003), and 20 Gy (V20, p= 0.001). The full course VMAT had the lowest volume of the lung irradiated by a dose of 30 Gy. There was no statistical significance observed regarding the protection of the heart among the three treatment schemes. No significant difference was observed regarding the dose to the volume of healthy tissue outside of PTV

**Table 2:** Target coverage comparison

*PTV*	*CRT*	*SD*	CRT+VMAT	*SD*	*VMAT*	*SD*	*p*
Dmax (cGy)	6432.2	259.0	6345.4	68.6	6256.8	154.2	0.11
Dmean (cGy)	5956.6	138.7	5971.0	32.9	5950.4	58.7	0.87
EUD (cGy)	5912.8	149.0	5958.5	35.0	5930.4	86.3	0.55
TCP	0.96	0.02	0.97	0.003	0.97	0.006	0.31
PTV 93^a^	95.3	7.3	99.9	0.1	99.2	1.3	0.05
PTV 95^a^	89.3	14.2	99.6	0.4	97.7	2.1	0.02
CI	0.5	0.2	0.6	0.1	0.7	0.1	0.005
CN	0.5	0.2	0.6	0.1	0.7	0.09	0.005
HI	0.998	0.007	0.999	0.003	0.999	0.001	0.40

a PTV_93 and PTV_95 are the percentage of the volumes of PTV covered by 93% and 95% of the prescription dose, respectively.

SD = standard deviation.

**Table 3:** OAR sparing comparison

	*CRT*	*SD*	*CRT+VMAT*	*SD*	*VMAT*	*SD*	*p*
Cord							
Dmax (cGy)	4293.4	157.6	4151.4	93.7	3832.5	272.5	<0.001
Dmean (cGy)	3002.1	459.6	2960.0	29.1	2396.2	881.8	0.07
EUD (cGy)	3832.2	98.6	3791.9	77.1	3270.1	288.6	<0.001
NTCP $\left(\times \;10^{-4}\right)$	86.5	20.8	78.3	13.1	26.4	16.2	<0.001
*Lung* Dmean (cGy)	1336.7	344.0	1234.1	300.0	1319.1	259.2	0.73
NTCP	0.03	0.1	0.01	0.04	0.01	0.01	0.56
V5	52.8	13.7	62.9	19.4	70.3	20.4	0.11
V10	40.7	11.8	36.6	10.1	55.6	16.3	0.007
V13	37.1	11.2	27.7	6.9	44.4	10.9	0.003
V20	30.7	9.4	18.8	5.2	21.9	2.4	0.001
V30	13.3	4.7	13.6	4.1	8.8	2.0	0.014
*Heart* Dmean (cGy)	1800.4	1370.6	1897.3	1431.3	1322.8	1036.5	0.57
EUD (cGy)	2845.4	1055.4	2956.5	1162.4	2127.6	1150.4	0.22
NTCP	0.06	0.1	0.07	0.1	0.004	0.007	0.26
V25	37.1	31.4	36.1	29.5	21.7	22.4	0.4
V30	33.6	29.5	34.2	28.8	15.5	15.6	0.2
V50	7.8	9.4	9.9	10.3	4.3	4.3	0.34
*Body* V5	49.3	12.7	58.3	15.3	65.2	18.7	0.09
V10	41.6	11.0	38.9	10.6	47.1	16.0	0.36
V15	37.6	10.3	30.3	8.0	31.4	11.5	0.24

## IV. DISCUSSION

IMRT has shown to be superior to CRT in target coverage and normal tissue sparing in the treatment of esophageal cancer.^(2)^ Studies have also indicated that VMAT is essentially equivalent to IMRT in the treatment of the esophagus from a dosimetric perspective.^(^ ^10^ ^,^ ^17^ ^)^ In the present study, a single‐arc VMAT boost plan following APPA conformal beams for upper thoracic esophageal cancer was demonstrated to be superior to the CRT in terms of better target coverage and the full course VMAT plan in terms of decreasing the lung volume irradiated by a low dose.

The cervical and upper esophageal areas lie in close proximity to the spinal cord. Drastic change in anatomical contours and diameters are often seen in upper thoracic esophageal cancer patients. The CTV of upper thoracic esophageal cancer usually includes the supraclavicular nodes at risk, and a part of the spinal cord may be projected inside the treatment field if lateral opposed beams or other off‐cord conformal beams are applied during the boost phase. Because of this geometric difficulty, the dose coverage of the PTV usually had to be sacrificed in order to spare the spinal cord within its tolerant dose (45 Gy). This target coverage sacrifice is observed in our study. Using VMAT boost instead of off‐cord conformal beams, the mean percent average target coverage of PTV (PTV_95) increased from 89.3 ± 14.2 to 99.6 ± 0.4 (p = 0.02). There was no significant difference (p = 0.87) in PTV_95 between CRT plus a sequential VMAT boost and the full course VMAT plan. The PTV_93 of CRT plus VMAT and full course of VMAT were also higher than that of CRT, but without statistical significance (p = 0.05). However, full course of VMAT plans demonstrated a higher CI (p = 0.01) and CN (p = 0.01) than did the other two treatment schemes, which is consistent with results of previous studies.^(^ ^2^ ^,^ ^9^ ^)^ No significant difference was observed for HI (p = 0.4).

The spinal cord is one important organ with strict dose limitation. CRT plus a sequential VMAT boost plan achieved a relatively lower spinal cord NTCP (78.3 ± 13.1) than did CRT plans (86.5 ± 20.8), but no statistically significant differences were observed (p = 0.54). A clear spinal cord sparing advantage was found in full course of VMAT plans over CRT plans and CRT plus a sequential VMAT boost. The average maximum dose and EUD of the full course VMAT plans were about 461 cGy and 552 cGy less than those CRT plans, respectively (p < 0.001). The NTCP of the full course VMAT plans was nearly three times less than (p < 0.001) those of CRT and CRT plus a sequential VMAT boost. However, the maximum spinal cord doses in all three treatment schemes were within the tolerance level.

Pulmonary complications were another one of the major concerns associated with esophageal cancer radiotherapy. Several heterogeneous dosimetric parameters have been proposed to correlate with the incidence and severity of pneumonitis.^18^, ^19^, ^20^, ^21^ In this study, we compared these dosimetric parameters among these three planning schemes accordingly. There was no significant difference observed regarding the mean lung dose and NTCP among the three treatment schemes. The mean dose of the lung was considered to be the most useful predictor of radiation pneumonitis in thoracic tumor radiation in a study by Kwa et al.^(18)^ On average, CRT plus off‐cord conformal beams showed the lowest V5, but this difference was not statistically significant (p = 0.11). V5 was also considered a strong indicator of pneumonitis in one postoperative chemoradiation study.^(19)^ CRT plus a sequential VMAT boost demonstrated clear advantages in V10 (p = 0.007) and V13 (p = 0.003) compared with CRT plus off‐cord conformal beams and full course of VMAT plans. V10 and V13 were also suggested to be strong indicators for pulmonary pneumonitis in a retrospective study.^(20)^ CRT plus a sequential VMAT boost also showed the lowest value (p = 0.001) in lung V20, which was also found to be strongly correlated with the severity of pneumonitis.^(20)^ Full course of VMAT plans presented a smallest value in V30, which also correlated to radiation pneumonitis in a study by Graham et al.^(21)^ Although there is no single dosimetric parameter that is agreed to be the best predictor of radiation pneumonitis following radiotherapy, it is believed that lower volumes of the lung receiving intermediate and low‐dose exposures are associated with better prognosis.^(5)^ CRT plus a sequential VMAT boost demonstrated clear advantage in most of the lung dosimetric parameters in this study.

The lung sparing advantage associated with CRT plus a sequential VMAT boost is similar to the hybrid IMRT technique used in the study by Mayo et al.,^(5)^ in which static conformal beams were concurrently combined with IMRT beams in the treatment of lung and esophageal cancer patients and were compared with CRT and IMRT. This result indicates that hybrid VMAT with APPA conformal beams concurrently combined with a single‐arc VMAT beam may be a potential technique in the treatment of esophageal cancer patients. However, this hybrid technique applying arc beam and conformal beams together in one fraction could increase the complexity of the planning process and beam delivery. Thus, applying VMAT only as the second boost phase is much more applicable and acceptable.

Full course of IMRT plans have been demonstrated to produce more conformal high‐dose distributions to the PTV at the cost of low doses to more normal lung tissue.^(22)^ Our study with full course of VMAT plans presented similar results. The full course VMAT plan achieved better target coverage and greater conformal dose distribution, as indicated by CI and CN, but a greater lung volume irradiated by the intermediate dose. Applying VMAT as only the second boost phase can reduce the volume of lung radiation compared to both CRT plus off‐cord conformal beams and the full course VMAT plan. At the same time, this method also improved the target coverage compared to CRT.

One potential problem with the full course VMAT plan is the increased volume of healthy tissue receiving low‐dose radiation outside the PTV. In this study, conformal radiotherapy achieved the lowest V5 in healthy tissue, although with no statistical significance (p = 0.09). There was also no statistical significance for healthy tissue in V10 and V15. This result differs a little from that of the study of Vivekanandan et al.^(9)^ They compared RapidArc (RA), CRT, and IMRT for ten esophageal cancer patients and found that V10 and integral dose to healthy tissue were similar for all the techniques; however, the RA plans resulted in a reduced low‐level dose bath (15–20 Gy) in the range of 14%‐16%, compared to the IMRT plans. The whole course VMAT plans demonstrated lower values on the heart parameters compared to CRT and CRT plus VMAT, but no significant differences (all p > 0.05) were observed regarding these parameters. This could also due to that heart was not involved in the inverse optimization.

## V. CONCLUSIONS

Using APPA conformal beams plus a sequential VMAT boost for upper thoracic esophageal cancer can improve the target coverage compared to using CRT plus an off‐cord conformal beams boost, while concurrently reducing the volume of the lung irradiated by the intermediate dose. CRT combined with a sequential VMAT boost plan may be a promising treatment technique for upper thoracic esophageal cancer patients.

## ACKNOWLEDGMENTS

The study was supported by Wenzhou Science and Technology Bureau Funding (Y20120137) and the Scientific Research Foundation for the Returned Overseas Chinese Scholars (604090656/037).

## Supporting information

Supplementary Material

## References

[1] Bradley JD and Muti S . Carcinoma of the esophagus. In: Levitt SH, Purdy JA, Perez CA, Vijayakumar S, editors. Technical basis of radiation therapy: practical clinical applications, 4th revised edition Berlin: Springer; 2008: p.511–24.

[2] Fenkell L , Kaminsky I , Breen S , Huang S , Prooijen MV , Ringash J . Dosimetric comparison of IMRT vs. 3D conformal radiotherapy in the treatment of cancer of the cervical esophagus. Radiother Oncol. 2008;89(3):287–91. 18789828 10.1016/j.radonc.2008.08.008

[3] Palta JR , Deye JA , Ibbott GS , Purdy JA , Urie M . Credentialing of institutions for IMRT in the clinical trials. Int J Radiat Oncol Biol Phys. 2004;59(4):1257–59. 15234063 10.1016/j.ijrobp.2004.03.007

[4] Advanced Technology Consortium. NCI guidelines on the use of IMRT in clinical trials. Accessed January 2007. Available from: http://atc.wustl.edu/home/NCI/NCI_IMRT_Guidelines.html

[5] Mayo CS , Urie MM , Fitzgerald TJ , Ding L , Lo YC , Bogdanov M . Hybrid IMRT for treatment of cancers of the lung and esophagus. Int J Radiat Oncol Biol Phys. 2008;71(5):1408–18. 18262730 10.1016/j.ijrobp.2007.12.008

[6] Bertelsen A , Hansen CR , Johansen J , Brink C . Single arc volumetric modulated arc therapy of head and neck cancer. Radiother Oncol. 2010;95(2):142–48. 20188427 10.1016/j.radonc.2010.01.011

[7] Shaffer R , Morris WJ , Moiseenko V , et al. Volumetric modulated arc therapy and conventional intensity‐modulated radiotherapy for simultaneous maximal intraprostatic boost: a planning comparison study. Clin Oncol. 2009;21(5):401–07. 10.1016/j.clon.2009.01.014 19268554

[8] Wolff D , Stierler F , Welzel G , et al. Volumetric intensity modulated arc therapy (VMAT) vs. serial tomotherapy, step‐ and‐shot IMRT and 3D‐conformal RT for treatment of prostate cancer. Radiother Oncol. 2009;93(2):226–33. 19765846 10.1016/j.radonc.2009.08.011

[9] Vivekanandan N , Sriram P , Kumar S , Bhuvaneswari N , and Saranya K , Volumetric modulated arc radiotherapy for esophageal cancer. Med Dosim. 2012;37(1):108–13. 21940159 10.1016/j.meddos.2011.01.008

[10] Van Benthuysen L , Hales L , Podgorsak MB . Volumetric modulated arc therapy vs. IMRT for the treatment of distal esophageal cancer. Med Dosim. 2011;36(4):404–09. 21377864 10.1016/j.meddos.2010.09.009

[11] Radiation Therapy Oncology Group. RTOG 0436 A phase III trial evaluating the addition of cetuximab to paclitaxel, cisplatin, and radiation for patients with esophageal cancer who are treated without surgery. Version date: 05/03/2012. Available from: http://www.rtog.org/ClinicalTrials/ProtocolTable/StudyDetails.aspx?study=0436

[12] Wambersie A and Landber T . ICRU Report 62: Prescribing, recording, and reporting photon beam therapy. (Supplement to ICRU Report 50). Bethesda: ICRU; 1999.

[13] Lomax NJ and Scheib SG . Quantifying the degree of conformity in radiosurgery treatment planning. Int J Radiat Oncol Biol Phys. 2003;55(5):1409–19. 12654454 10.1016/s0360-3016(02)04599-6

[14] van't Riet A , Mak AC , Moerland MA , Elders LH , van der Zee W . A conformation number to quantify the degree of conformity in brachytherapy and external beam irradiation: application to the prostate. Int J Radiat Oncol Biol Phys. 1997;37(3):731–36. 9112473 10.1016/s0360-3016(96)00601-3

[15] Niemierko A and Goiten M . Modeling of normal tissue response to radiation: the critical volume model. Int J Radiat Oncol Biol Phys. 1993;25(1):135–45. 8416870 10.1016/0360-3016(93)90156-p

[16] Okunieff P , Morgan D , Niemierko A , Suit HD . Radiation dose‐response of human tumors. Int J Radiat Oncol Biol Phys. 1995;32(4):1227–37. 7607946 10.1016/0360-3016(94)00475-z

[17] Hawkins MA , Bedford JL , Warrington AP , Tait DM . Volumetric modulated arc therapy planning for distal oesophageal malignancies. Br J Radiol. 2012;85(1009):44–52. 21427179 10.1259/bjr/25428720 PMC3473937

[18] Kwa SL , Lebesque JV , Theuws JC , et al. Radiation pneumonitis as a function of mean lung dose: an analysis of pooled data of 540 patients. Int J Radiat Oncol Biol Phys. 1998;42(1):1–9. 9747813 10.1016/s0360-3016(98)00196-5

[19] Wang SL , Liao Z , Vaporciyan AA , et al. Investigation of clinical and dosimetric factors associated with postoperative pulmonary complications in esophageal cancer patients treated with concurrent chemoradiotherapy followed by surgery. Int J Radiat Oncol Biol Phys. 2006;64(3):692–99. 16242257 10.1016/j.ijrobp.2005.08.002

[20] Lee HK , Vaporciyan AA , Cox JD , et al. Postoperative pulmonary complications after preoperative chemoradiation for esophageal carcinoma: correlation with pulmonary dose‐volume histogram parameters. Int J Radiat Oncol Biol Phys. 2003;57(5):1317–22. 14630268 10.1016/s0360-3016(03)01373-7

[21] Graham MV , Purdy JA , Emami B , et al. Clinical dose‐volume histogram analysis for pneumonitis after 3D treatment for non‐small cell lung cancer (NSCLC). Int J Radiat Oncol Biol Phys. 1999;45(2):323–29. 10487552 10.1016/s0360-3016(99)00183-2

[22] Chapet O , Fraass BA , Ten Haken RK . Multiple fields may offer better esophagus sparing without increased probability of lung toxicity in optimized IMRT of lung tumors. Int J Radiat Oncol Biol Phys. 2006;65(1):255–65. 16618580 10.1016/j.ijrobp.2005.12.028

