# Supplementary material for: CRT combined with a sequential VMAT boost in the treatment of upper thoracic esophageal cancer
Source: J Appl Clin Med Phys. 2013 Sep 6;14(5):153–61. doi: 10.1120/jacmp.v14i5.4325 (PMC5714557; doi:10.1120/jacmp.v14i5.4325)
Supplement: Supplementary file 1 — Supplementary Material [file ACM2-14-153-s001.docx]

**CRT combined with VMAT as the second boost phase in the treatment of upper thoracic esophageal cancer**

**Xiance Jin, Jinling Yi, Yongqiang Zhou, Huawei Yan, Ce Han, Congying Xie**Radiotherapy and *Chemotherapy Department of the 1^st^ Affiliated Hospital of Wenzhou Medical College, Wenzhou, China*

Corresponding author: Congying Xie, Ph.D

Radiotherapy and Chemotherapy Department of the 1^st^ Affiliated Hospital of Wenzhou Medical College

No.2 Fuxue Lane, Wenzhou, China, 325000

Phone: 0086-577-88069316, Fax: 0086-577-88069370

Email: [*billy07@wzhospital.cn*](mailto:xiecy@wzhospital.cn)

Running title: boost VMAT phase for upper esophageal cancer

Conflict statement: There was no actual or potential conflict of interest for this paper
